# Precision Connectivity in Arthritis Pain with Permutation and Network Analysis: A Key Step Toward Clinical Application

**DOI:** 10.21203/rs.3.rs-6915471/v1

**Published:** 2025-09-03

**Authors:** Belfin Robinson, Emilio G. Cediel, William Reuther, Aryan Kodali, Ellora Srabani, Olivia Leggio, Vibhor Krishna, Varina L. Boerwinkle

**Affiliations:** University of North Carolina at Chapel Hill; University of North Carolina at Chapel Hill; University of North Carolina at Chapel Hill; University of North Carolina at Chapel Hill; University of North Carolina at Chapel Hill; University of North Carolina at Chapel Hill; University of North Carolina at Chapel Hill; University of North Carolina at Chapel Hill

**Keywords:** rs-fMRI, chronic pain, osteoarthritis, seed analysis, permutation, network analysis

## Abstract

**Objective:**

This study seeks to identify brain regions with atypical neural connectivity in individuals suffering from arthritis-related chronic pain, compared to healthy controls, using resting-state functional magnetic resonance imaging (rs-fMRI).

**Methods:**

A seed-based connectivity analysis was conducted between the known pain-related regions of interest (ROIs), derived from the MNI (n = 76) and the Automated Anatomical Labeling (AAL) whole brain atlas (n = 116). We examined the connectivity differences in a cohort of 56 osteoarthritis patients and 20 healthy controls. Connectivity matrices were compared using permutation tests corrected for multiple comparisons, identifying statistically significant differences (p < 0.05). Subsequent network analysis resulted in hub scores, identifying the most central and influential brain regions within the altered connectivity network in patients experiencing pain.

**Results:**

The most significant atypical neural connections in osteoarthritis patients were identified in the cingulate gyrus, insula, inferior parietal lobe, and thalamus, with notable involvement of the occipital lobe, postcentral gyrus, inferior frontal gyrus, orbitofrontal cortex, temporal lobe, hippocampus, and basal ganglia. The thalamus, cingulate gyrus, and insula emerged as key hubs in the chronic pain network, reflecting disrupted sensory, emotional, and cognitive pain processing. No significant connectivity differences were found in the brainstem, cerebellum, superior parietal lobe, precentral gyrus, superior and middle frontal gyri, or amygdala.

**Conclusion:**

Our data-driven approach reveals specific neural connectivity disruptions in OA, highlighting connections between the cingulate gyrus, temporal lobe, and thalamus. These findings identify specific network disruptions in OA-related pain, offering insight into altered brain connectivity and potential avenues for targeted interventions.

## 1. Introduction

Individual patient-level advancements in data-driven resting-state functional magnetic resonance imaging (rs-fMRI) approaches have significantly enhanced the diagnosis and management of neurological disorders, by avoiding the assumption that hypothesis driven locations of abnormal brain function are correct for all patients. For example, in epilepsy [1–3], independent component analysis (ICA) has been instrumental in localizing seizure networks, which closely correspond to those identified by stereo-electroencephalography (SEEG) and confirmed by surgical outcomes [4]. Similarly, data-driven methods in movement disorders and coma have also contributed to precision-based clinical strategies [5, 6]. The success of these approaches suggests that similar methodologies could be applied to chronic pain, which is often recalcentric to standard therapy, and is the focus of this work.

Previous hypothesis-driven rs-fMRI studies have identified various brain regions associated with pain processing [7–9]. Key areas such as the thalamus, insula, anterior cingulate cortex, and prefrontal cortex have been consistently implicated in the perception and modulation of pain [10–12]. These regions are thought to be central to the brain’s pain network, orchestrating the complex experience of chronic pain in conditions such as osteoarthritis. Also, prior groupwise seed-based rs-fMRI analysis in osteoarthritis patients has been explored to distinguish between healthy controls and osteoarthritis patients, and the impact of treatments such as acupuncture [13, 14]. Recently, a summary of the brain-wide pain-related seed regions was published by Dai et al. [15], which may be useful for precision-based region brain-wide region of interest queries in chronic pain.

However, while there has been some progress from the hypothesis-based approach in rs-fMRI in chronic pain, the data-driven approaches, may be more likely to lead to more comprehensive precision based and clinically applicable information, as found in the examples of epilepsy and movement disorders.

For example, artificial intelligence (AI) chronic pain studies [9, 16, 17] utilized deep learning algorithms on rs-fMRI identify additional regions in chronic pain from osteoarthritis, such as the default mode network (DMN), salience network, central executive network, and sensorimotor network. Specifically, alterations in connectivity are observed in the prefrontal cortex, occipital lobe, superior frontal gyrus, insula, thalamus, and anterior cingulate cortex. However, despite the potential power of AI, these models face three significant challenges in pain related connectivity clinical applications, due to the following issues: (1) The primary issue is the lack of comprehensive datasets, limiting AI predictions’ robustness. [18] link pain catastrophizing to increased connectivity between the dorsolateral prefrontal cortex (DLPFC) and pain areas, emphasizing the need for varied data. The DLPFC is involved in multiple executive functions like working memory, attention, task switching, and emotional regulation, and interacts with regions like the amygdala [19]. Despite being linked to intelligence and speech, its role in these areas is not fully understood. Data scarcity risks biased AI models in pain prediction. (2) Additionally, variability in individual pain related rs-fMRI data presents another significant challenge. [20] demonstrated that acupuncture enhanced functional connectivity between the dorsal raphe nucleus (RPN) and other brain regions in knee osteoarthritis patients, but individual differences in brain response complicated the analysis, especially with smaller patient samples. Each patient’s unique brain patterns and responses to chronic pain make it challenging to develop AI models that consistently account for these variations. (3) Translating research findings into practical bedside solutions also remains limited. [21] found that stronger connectivity between the amygdala and cortical regions involved in sensory and motor responses was associated with greater pain facilitation by negative emotions, indicating that emotional states significantly influence chronic pain processing. This complexity contributes to the difficulty of using AI models to predict clinically useful biomarkers.

Thus, AI models in chronic pain research are not yet sufficiently developed for reliable clinical application, due to the need to incorporate individual variability, and enhancing the translational aspects of AI research are critical for improving the utility of these models for patient care.

To address these challenges, the data-driven approaches of permutation analysis **proposed for the current study**, offers a promising solution [22]. Permutation testing is advantageous in scenarios with limited data, as it allows for robust statistical analysis without relying on assumptions about data distribution [23, 24]. This method helps identify atypical brain connectivity patterns by comparing connectivity matrices, thus overcoming the limitations posed by small sample sizes.

In this study, we define “atypical connections” as functional connectivity links between brain regions in osteoarthritis patients that significantly deviate from the distribution observed in healthy controls. These connections were identified through permutation testing with correction for multiple comparisons, representing patient-specific deviations from normative connectivity patterns. By focusing on these individualized abnormalities, our analysis aims to uncover patterns of neural dysregulation associated with chronic pain in osteoarthritis. The overarching goal of this study is to develop a precision-based approach to identify an individual’s most atypically interconnected brain, serving as a critical first step toward the long-term objective of creating personalized therapeutic strategies. Building on this, we explored atypical brain connectivity related to chronic pain in osteoarthritis patients compared to healthy controls by applying permutation analysis to brain-wide functional connectivity, focusing on pain-related regions summarized by [15]. Additionally, we conducted a group-level analysis to validate the precision method by comparing our findings to those reported in previous studies.

In summary, this research seeks to advance the field of neuroinformatics within the context of osteoarthritis chronic pain, providing insights that could ultimately inform more effective diagnostic and precision-guided therapeutic interventions.

## 2. Methods

**Figure 1:**
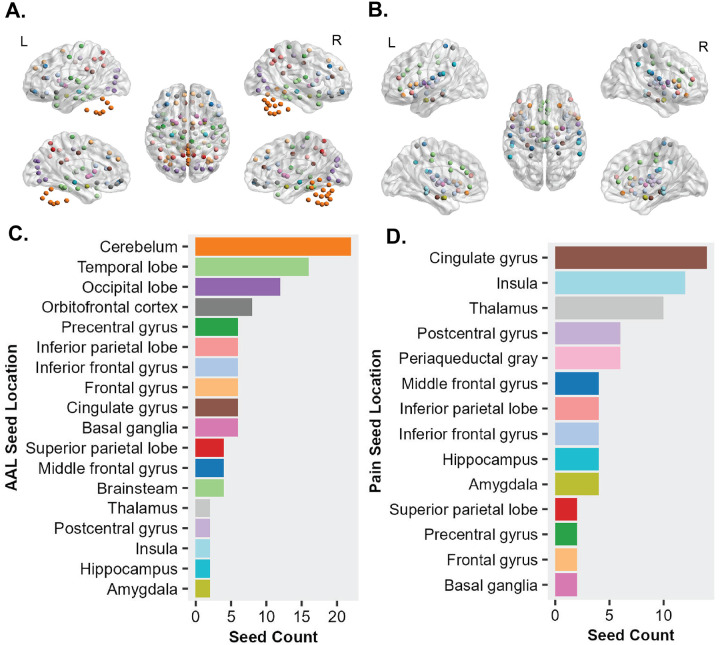
Regions of Interest (ROIs) for Resting-State fMRI Seed Analysis. (A.) The AAL atlas depicting 116 parcellated brain region ROI points. (B.) Locations of seed points in the brain associated with pain perception, based on Dai et al., 2018. (C.) Location-based grouping of whole brain AAL ROIs, with colors in figure 1A indicating the same location groups. (D.) Location-based grouping of pain seeds, with colors in figure 1B indicating the same location groups.

**Figure 2 A.**
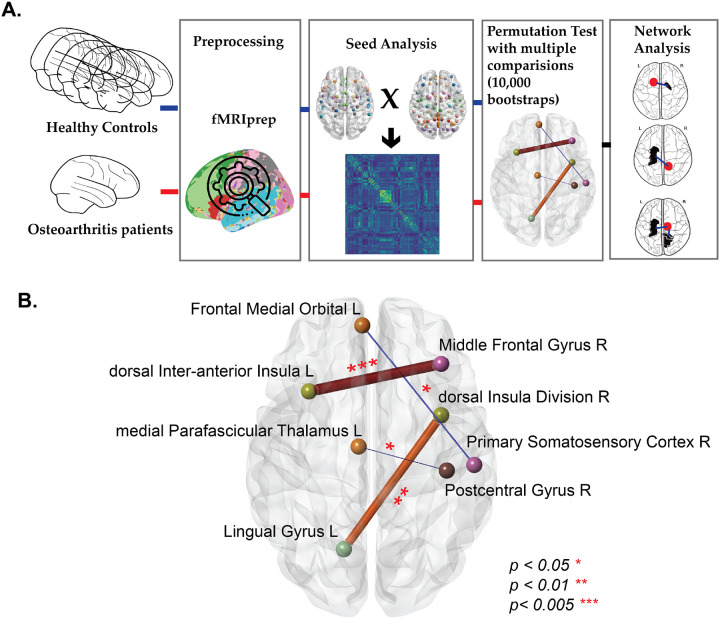
Study Analysis Flow Summary. Methodological framework for analyzing atypical functional connections between osteoarthritis patients and healthy controls. B. This diagram displays connections that are significantly different (p<0.05) in one patient when compared to a set of healthy controls.

This study utilizes seed-based connectivity analysis to investigate the differences in neural connectivity between osteoarthritis patients and healthy controls. By focusing on pain-related ROIs (Fig. 1A, Fig. 1C) and employing the Automated Anatomical Labeling (AAL) atlas for whole-brain region identification (Fig. 1B, Fig. 1D), we aim to offer a detailed map of atypical connectivity that may contribute to pain perception and modulation in osteoarthritis. The analysis was conducted using publicly available open rs-fMRI data [25], which was preprocessed through the fMRIPrep pipeline to ensure consistency and accuracy. Seed-based functional connectivity was assessed between pain-related brain regions and other areas defined by the brain atlas, with permutation analysis employed to validate the significance of observed connectivity differences. The use of permutation tests with a substantial number of bootstraps ensures robustness against the issue of multiple comparisons, a common challenge in neuroimaging studies. Group analyses were also conducted to identify common atypical connections across patients. This methodological framework (Fig. 2) not only enhances our understanding of osteoarthritis but also offers a template for studying other diseases by providing a reproducible approach to mapping atypical brain connectivity.

### 2.1. Preprocessing

The preprocessing of the fMRI data was conducted using the standard fMRIPrep pipeline (Esteban et al., 2019). This pipeline executes a series of preprocessing steps to ensure consistency and reproducibility across the dataset. Initially, anatomical T1-weighted (T1w) images were skull-stripped and segmented into gray matter, white matter, and cerebrospinal fluid. Subsequently, the functional images underwent motion correction, slice-timing correction, and alignment to the individual T1-weighted images. Normalization to a standard template (MNI space) was performed, followed by spatial smoothing. Confound regressors, including motion parameters, cerebrospinal fluid, and white matter signals, were extracted. Regression on six motion parameters was performed to reduce noise and artifacts. The initial five volumes from the functional images were discarded to allow for signal stabilization. The final preprocessed images were visually inspected for quality assurance.

### 2.2. Seed-based Connectivity Analysis

Seed analysis is a technique used to explore the temporal correlation between a predefined seed region and the whole brain regions. This method provides valuable insights into the functional connectivity within the brain, particularly how different regions interact within a specific network [26]. In the context of this research, seed analysis was employed to understand the pain functional network.

For this study, we utilized 76 pain-related coordinates in MNI space as seed regions, based on research by [15]. These seeds were strategically chosen to represent key areas implicated in pain perception and processing (Fig. 1B). The selection of seed points for analysis includes brain locations such as the insula (12 seeds), amygdala (4 seeds), thalamus (10 seeds), among others, based on the literature (Fig. 1D). The (x,y,z) coordinates of the seed and the location of the seed has been given in supplementary information (**Table S1**). By analyzing the temporal correlations between these seed regions and the rest of the brain, we aimed to identify connectivity patterns characteristic of osteoarthritis pain. For comprehensive whole-brain analysis, we employed the AAL atlas, which parcels the brain into 116 regions (Fig. 1A). This atlas facilitates detailed examinations of functional connectivity across various brain regions (Fig. 1C). The (x, y, z) coordinates of the AAL ROI and the location of the ROIs has been given in the supplementary information (**Table S2**). By correlating the activity in predefined seed regions with all other regions defined by the AAL atlas, we generated a 76×116 matrix for each patient and control subject. This matrix represents the correlations between the 76 seed regions and the 116 brain regions, providing a comprehensive view of the brain’s functional connectivity as outlined by the AAL atlas.

### 2.3. Statistical Analysis: Permutation Testing for Functional Connectivity Analysis

In this study, we employed a permutation testing approach to assess the statistical significance of differences in functional connectivity between the individual patient and the group of control subjects. Permutation tests are a non-parametric method of statistical inference that provide p-values without assuming a normal distribution, making them particularly suitable for fMRI data [27].

The functional connectivity data were structured as 76×116 matrices for each subject. Controls and patients were represented as a 3D array (20 × 76 × 116) and a 2D array (76 × 116), respectively. For each specific ROI-to-ROI connection, we computed the absolute difference in mean connectivity values between the controls and patient.

Data from both groups were concatenated, and 10,000 permutations were conducted by randomly shuffling the labels while maintaining original group sizes. For each permutation, the mean difference was recalculated. The observed mean difference was then compared against the distribution of permuted mean differences to calculate p-values, reflecting the proportion of permuted mean differences that were as extreme or more extreme than the observed mean difference. This non-parametric approach determines the statistical significance of connectivity differences without relying on parametric assumptions.

For each ROI-to-ROI connection, the p-value was the proportion of permutations with a mean difference as large as or larger than the observed difference. To control for type I error given the numerous comparisons, we applied False Discovery Rate (FDR) correction, keeping the false positives proportion at α = 0.05. These connections are represented as nodes (ROIs) and edges (connections between nodes) in Fig. 2B. The plot illustrates connections between the pain ROI and other atlas regions in the brain. The thickness of the edges indicates the level of significance, with thicker edges representing higher significance. Additionally, the size of each node reflects the frequency with which the ROI appears in the patient’s atypical network, providing insight into the most affected brain regions for that specific patient.

Although permutation testing has been used to assess statistical significance in group differences, we evaluated potential confounders including age, sex, and mean framewise displacement and found no significant differences between OA patients and healthy controls on these variables (all *p* > 0.1).

### 2.4. Network Analysis

Network analysis is a robust method for investigating the structural nuances of complex networks by identifying and comparing recurring subgraph patterns, known as motifs. These motifs are pivotal in revealing the underlying architecture and functionality of various networks, ranging from biological to social systems [28]. In practical terms, network analysis is utilized to discern the roles and relationships within networks, offering insights into operational mechanisms in biological contexts or communication patterns in social structures [29, 30]. For example, by comparing brain network motifs of healthy individuals against those with neurological disorders, researchers can pinpoint disruptions in connectivity that might relate to specific diseases, such as osteoarthritis.

The methodology behind network analysis involves several critical steps. Our methodology involves several key steps. In this study, we focused on identifying nodes that recur across patients with pain, based on both the AAL atlas and specific pain-related ROIs. We then calculated the connections shared across these patients and identified the recurring motifs, allowing us to detect patterns in the altered connectivity associated with chronic pain.

## 3. Results

The data obtained from the OpenfMRI database (accession number ds000208) includes 20 healthy controls and 56 osteoarthritis (OA) pain patients. The original study [25] comprised three separate studies designed to evaluate the predictability of brain connectivity changes in response to placebo and active drug treatments.

Study 1 (n = 17) involved single-blind placebo administration over two weeks, followed by a two-week washout period. Study 2 (n = 39) was a double-blind randomized trial where participants were assigned to either placebo or duloxetine treatment for three months. Study 3 (n = 42) served as a no-treatment control group, tracking knee OA pain changes over three months without any interventions or brain scans.

For our analysis, we combined data from all three studies into a single cohort, disregarding treatment distinctions, and included each patient’s current pain status at the time of the scan, based on demographic information available in the database. This approach enabled us to conduct a cross-sectional comparison between OA patients and healthy controls to identify atypical brain connectivity patterns associated with chronic pain, irrespective of specific clinical trial conditions. This method provides a comprehensive overview of baseline brain connectivity alterations in OA patients. Table 1 summarizes the demographic characteristics of the combined cohort, contrasting patient data with that of healthy controls.

### 3.1. ROI-based (node) osteoarthritis patient analysis

In this study, we investigated atypical neural connections in patients suffering from osteoarthritis pain. An example subject-level atypical connections is shown in (Fig. 2B, **Figure S1a**). Utilizing a permutation-based method, we identified 293 unique atypical connections across. **Figure S1b** shows the distribution of these atypical connections across individual patients. At least 92.8% of patients exhibited at least one atypical connection. The number of atypical connections per patient ranged from zero to 27. Ten of the 56 patients have exactly three atypical connections. However, as depicted in the density plot, (**yellow overlay Figure S1b**) the most prevalent number of atypical connections per patient is between 5 and 7.

Further analysis revealed specific ROIs that consistently exhibited atypical connectivity. Two ROI sets were used in our seed-based analysis, the AAL ROIs (atlas segmented regions) and the Pain Seed ROIs (single coordinate). Therefore, the node findings are described by each ROIs set. The top 15 AAL ROIs were recurrent in 56 osteoarthritis patients, (**Figures S2a and S2b**). This region including the left middle cingulate, right lingual gyrus, right pallidum, left supramarginal gyrus, left thalamus, and right Rolandic operculum as significantly atypical – as atypical **in more than 10 patients**.

In contrast, several other regions did not show statistically significant connectivity across the cohort. These included the left caudate, left and right cerebellar lobes (notably in regions such as Crus 1, Crus 2, 7b, and 10), various areas of the vermis (including Vermis 9, 10, and Vermis 1–2), the right anterior cingulate gyrus, bilateral supplementary motor areas, the left and right superior medial frontal gyri, the left hippocampus, and the right inferior frontal orbital region. Additional areas such as the precentral gyrus, precuneus, and regions of the olfactory and temporal poles were also evaluated but did not demonstrate significant atypical connectivity. The absence of statistical significance in these regions suggests that their role in pain processing in osteoarthritis may be more limited or variable across individuals.

Similarly, analysis of the Pain Seed ROIs identified several critical areas showing common atypical connections including: the right posterior midcingulate cortex, left dorsal granular insula, left medial prefrontal thalamus, right and left caudal hippocampus, left dorsolateral putamen, left premotor thalamus, left caudal ventral anterior cingulate cortex, right caudal ventral posterior cingulate cortex, and left primary somatosensory cortex (trunk region). Each of these regions demonstrated atypical connectivity in over **ten instances** among the studied cohort of fifty-six osteoarthritis patients.

In addition to these findings, several other pain seed regions do not show atypical connections across the patient cohort. These regions include the right dorsolateral putamen, left and right rostroventral ventral anterior cingulate cortex, right subgenual dorsal anterior cingulate cortex, left orbitofrontal cortex, left opercular pars triangularis, left ventral pars triangularis, left ventral agranular insula, right dorsal granular insula, left ventral dorsolateral prefrontal cortex, right ventrolateral prefrontal cortex, right ventrolateral periaqueductal gray, right lateral periaqueductal gray, left and right dorsolateral periaqueductal gray, left and right primary somatosensory cortex (tongue and larynx regions), left and right primary motor cortex, right postcentral somatosensory association cortex, and the right caudal temporal thalamus.

**Figure 3:**
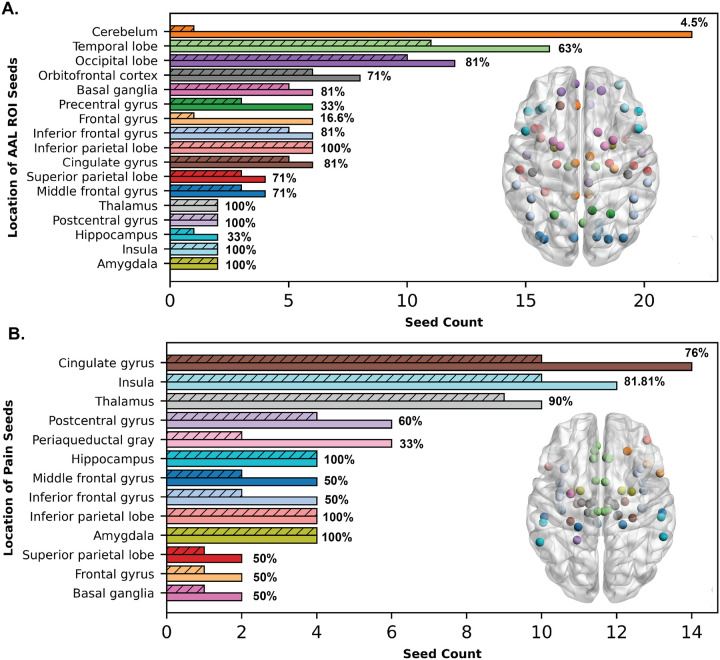
Atypical Connection Representation in Region Groups Before and After Analysis. (A) This panel illustrates the percentage representation of atypical connections identified through permutation analysis, relative to the total AAL atlas regions used in each group. Solid bars represent the initial atlas region groups used as ROIs for the analysis, while dotted bars indicate the representation of these regions in atypical connections identified post-analysis. The inner brain plot with nodes represents the AAL ROIs (color-coded based on their brain location) that are involved in atypical connections across the osteoarthritis patient cohort. (B) This panel shows the representation of pain seed nodes in the cohort, comparing their presence before (solid bars) and after (patterned bars) the analysis. The inner brain plot with nodes represents the pain seeds (color-coded based on their brain location) that are involved in atypical connections across the osteoarthritis patient cohort.

We conducted a systematic analysis of seed location distributions across a cohort of 56 osteoarthritis patients, comparing all seed regions to those exhibiting significant atypical neural connections (Fig. 3A). Of the seventy-six seed regions initially evaluated, only fifty-five were found to contribute to significant atypical connectivity. Additionally, we compared the total AAL ROIs employed with those that demonstrated atypical connectivity across the cohort. Out of the 116 AAL ROIs used, only 68 were identified as exhibiting atypical connectivity (Fig. 3B).

Notably, seeds from critical regions such as the inferior parietal lobe, inferior frontal lobe, and amygdala were recurrently identified across various atypical networks, underscoring their potential role in aberrant neural activities associated with osteoarthritis pain. In stark contrast, none of the seed regions from the precentral gyrus were represented in the atypical networks, suggesting a differential regional involvement in the disease pathology among OA patients.

### 3.2. ROI Connection-based (edges) osteoarthritis patient analysis

**Figure 4:**
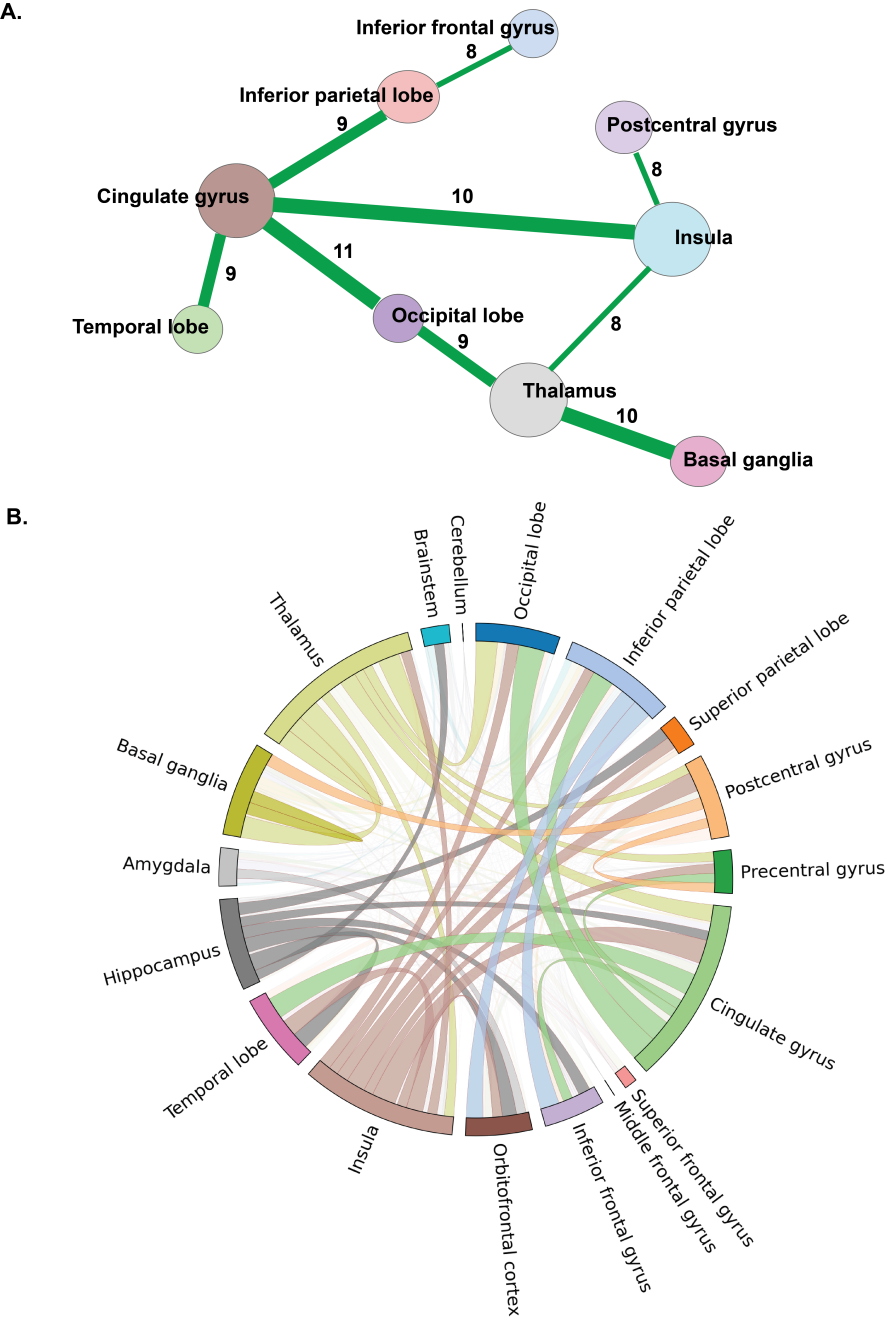
Atypical connections repeated more than 7 times across study subjects. (A.) The larger the node size, the more frequently the region is represented in atypical connections across the patient cohort. The width of the link and the weight in the link (numeral in black) represent the number of times this atypical connection is repeated across the patient cohort. (B.) **Atypical connections between different brain regions in study subjects.** This plot includes connections that occurred more than four times between regions. The cingulate gyrus, insula, and thalamus shared more connections with various other regions, whereas the middle frontal gyrus, superior frontal gyrus, and brainstem showed fewer connections with other regions.

Our analysis of atypical network connections between brain regions of interest (ROIs), as shown in Fig. 4A, employs a stringent threshold criterion: connections must appear in more than seven different patient assessments to be included in this subnetwork plot. The entire unfiltered network of locations, which includes all connections among atypical regions, is provided in **Figure S4**. This thresholding approach enhances the accuracy and reliability of visualization by focusing on consistently observed interactions to offer a clear metric of its frequency and significance across the cohort.

Regions such as the cingulate gyrus and occipital lobe exhibited atypical connections in 11 patients, The atypical connections observed were predominantly bilateral. The most frequently observed atypical functional connections were between the cingulate gyrus and the insula (10 patients) and between the thalamus and basal ganglia (10 patients), followed by altered connections between the cingulate gyrus and the parietal and temporal lobes, as well as between the thalamus and the insula and occipital lobes. These consistently observed altered connections suggest a role in affected neural network dynamics related to pain processing in osteoarthritis, particularly involving the Thalamus and the Cingulate Gyrus. The whole atypical network between brain location without thresholding has been illustrated in **Figure S4**.

Atypical connections between voxel-level and seed-level (ROI-level) are shown only if observed in more than three patients, with a threshold set for connections evident across these cases (**Figure S3**). Notably, the atypical connection between the right pallidum and left premotor thalamus was observed in eight patients. Similarly, atypical connections between the left parahippocampal and left caudal hippocampus, as well as between the left thalamus and left medial prefrontal thalamus, were each identified in seven patients. As outlined in (**Figure S3**), these atypical connections, evident in up to four patients, were thresholded to ensure clarity and reproducibility within the visualization. This methodological approach underscores the consistency and potential relevance of these neural pathways in the pathology of osteoarthritis pain.

To provide a higher-level view of atypical connectivity, ROIs derived from pain seeds and the AAL atlas were systematically categorized into 18 distinct region groups. This classification facilitated a detailed analysis of the atypical connectivity patterns among these groups, as depicted in Fig. 4B. The figure illustrates the atypical connections between various brain regions within the patient cohort, focusing on connections that were observed more than four times between regions, thereby depicting the consistency and significance of the identified connections.

To examine the clinical relevance of individual-level connectivity differences, we tested whether the strength of significant brain region-to-region connections correlated with pain-related clinical outcomes, specifically VAS pain reduction and WOMAC improvement scores. Age-related effects on pain reduction were further explored by stratifying correlations by gender (**Figure S5**), revealing a significant negative correlation between age and VAS pain reduction in females, but not in males. Among the connections identified as significantly different between chronic pain and control participants, only two showed a modest linear relationship with VAS pain reduction.

The first connection between the right inferior parietal lobe and the left inferior frontal gyrus was associated with VAS pain reduction (*R*^*2*^ = 0.10; linear fit: *y* = 8.71 + 67.87x), suggesting that increased connectivity may be modestly related to improved pain scores. The second connection between the left orbitofrontal cortex and the right hippocampus showed a slightly stronger relationship (*R*^*2*^ = 0.15; *y* = 3.93 + 91.92x). All other significant region-pair correlations with either VAS or WOMAC scores showed *R*^*2*^ values < 0.10, suggesting minimal predictive value. Full plots and model fits are provided in Supplementary **Figure S6**.

Our analysis revealed notable patterns of connectivity: the cingulate gyrus, insula, and thalamus emerged as prominently atypical hubs, exhibiting a higher number of atypical connections with multiple other regions. Conversely, regions such as the middle frontal gyrus, superior frontal gyrus, and brainstem showed fewer connections with other areas.

## 4. Discussion

This study presents a novel approach to understanding osteoarthritis (OA) pain by identifying individual-level atypical connectivity patterns in 56 OA patients, compared to healthy controls. Unlike previous studies that focused on group-level analyses, our patient-specific findings reveal clinical heterogeneity within OA, a condition often considered homogeneous. This individualized insight into brain connectivity advances our understanding of OA pain’s neural underpinnings and may guide future developments in personalized care. We acknowledge, however, that some degree of inter-individual variability in resting-state connectivity is expected even among healthy controls. Since the control group serves as the normative reference, future analyses should apply the same permutation-based method to controls to quantify the baseline prevalence of atypical connections, helping to clarify the specificity and pathological significance of observed deviations in OA patients.

Key regions consistently exhibiting atypical connectivity include the right posterior midcingulate cortex, left granular insula, left medial prefrontal thalamus, bilateral caudal hippocampus, left dorsolateral putamen, left premotor thalamus, and left primary somatosensory cortex. These areas are integral to the sensory, emotional, and cognitive processing of pain, aligning with established pain-related networks [31–33]. Our study’s identification of these specific brain regions at an individual level emphasizes the diversity in pain perception and processing across OA patients, underscoring the need for targeted, patient-specific treatments.

### 4.1. Key Regions in Pain Processing

The study identified 293 atypical neural connections, notably in the medial and dorsolateral prefrontal cortices, cingulate cortex, insula, thalamus, hippocampus, and putamen, which are critical for modulating sensory, emotional, and cognitive responses to pain (Bräscher et al., 2016; Misra & Coombes, 2015). Additionally, certain regions—including the left middle cingulate, right lingual gyrus, right pallidum, and left supramarginal gyrus—displayed atypical connectivity in over 10 patients, indicating their potential involvement in pain comorbidities like anxiety and depression (Biggs et al., 2020). This patient-specific approach could reveal underlying mechanisms that broader group analyses may overlook, highlighting the unique neural responses to pain in each OA patient.

### 4.2. Atypical Connectivity and Abnormal Neural Communication

Core pain-processing regions, including the hippocampus, amygdala, insula, and thalamus, showed 100% representation of atypical connections across patients. Such widespread disruptions in sensory-emotional networks reinforce their roles in chronic pain conditions (Mutso et al., 2014; Simons et al., 2014; Barroso et al., 2021). In OA, the hippocampus, with altered connectivity patterns, may contribute to pain anticipation and memory recall, while atypical insular connectivity may enhance pain perception by integrating sensory and emotional inputs (Labrakakis, 2023; Wiech et al., 2014). These findings emphasize how personalized mapping of atypical connectivity could inform the development of therapies targeting specific regions to manage both the sensory and emotional components of pain.

### 4.3. Abnormal Two-Way Connectivity in Chronic Pain Networks

Significant atypical two-way connectivity patterns between the cingulate gyrus, thalamus, and insula were observed in 19%, 18%, and 14% of chronic pain patients, respectively. These abnormal connections disrupt reciprocal communication pathways that support pain integration (Peyron et al., 2000; De Ridder et al., 2021). The thalamus exhibited atypical connectivity with the basal ganglia (18%), occipital lobe (16%), insula (14%), and itself (12%), reflecting disruptions in sensory, motor, and integrative networks relevant to chronic pain (Aoe et al., 2024; Borsook et al., 2010). Our findings suggest these regions as potential intervention targets, particularly given their involvement in integrating sensory and motor information.

### 4.4. Network Hub Analysis

The network analysis identified the thalamus as the most central hub in chronic OA pain-related networks, with a hub score of 1.00, reinforcing its established role in sensory relay and pain modulation. Here, hub score refers to degree centrality, quantifying how many atypical connections a region maintains across the network. Other high-scoring regions included the cingulate gyrus (hub score = 0.85) and the insula (0.73), both of which are integrally involved in the affective and interoceptive dimensions of pain [34, 35]. Additional hubs—such as the occipital lobe (0.57), basal ganglia (0.53), and inferior parietal lobe (0.50) highlight that chronic OA pain is associated with distributed network disruptions spanning sensory, motor, and attentional systems [36, 37].

While the thalamus, cingulate, and insula are also activated in acute pain states, their role as central hubs in the resting-state networks of OA patients absent of any experimental pain stimulus suggests persistent, trait-like alterations rather than transient nociceptive activation. These findings provide mechanistic insight into chronic pain processing and may support future network-targeted interventions aimed at restoring functional balance in these key regions.

### 4.5. Implications for OA Pain and Neural Alterations

This study underlines the importance of atypical connectivity patterns in OA, a prevalent condition marked by joint pain and neural alterations. Brain regions such as the prefrontal cortex, anterior cingulate cortex, insula, thalamus, and hippocampus play essential roles in pain modulation, emotional processing, and memory. Altered connectivity within these regions reflects the complex experience of OA pain and its impact on cognitive and emotional health, suggesting therapeutic targets for symptom relief [38–40]. Since we statistically identified abnormal brain regions in each patient compared to healthy controls and subsequently analyzed these regions as a group, we verified that the identified abnormalities are consistent markers of OA pain. These findings highlight promising areas for future research to explore interventions that address the unique neural connectivity profiles of each OA patient.

### 4.6. Modest Clinical Correlations with Altered Network Connectivity

While our primary analysis focused on individual-level connectivity differences between chronic pain patients and healthy controls, we also examined whether these connectivity patterns were meaningfully related to clinical outcomes. Of the significantly altered connections, only two showed modest associations with VAS pain reduction: the right inferior parietal lobe and the left inferior frontal gyrus (*R*^*2*^ = 0.10), and the left orbitofrontal cortex and the right hippocampus (*R*^*2*^ = 0.15). In contrast, no meaningful associations were found between altered connectivity and WOMAC pain reduction scores (*R*^*2*^ < 0.10 for all region pairs), suggesting that functional connectivity may be more closely related to subjective pain intensity than to broader functional improvement as measured by WOMAC. These findings suggest that while resting-state connectivity alterations are evident at the individual level, only a limited subset may directly reflect pain relief or therapeutic response. This aligns with prior work indicating that neural signatures of chronic pain are complex and often influenced by non-pain-specific factors such as mood, attention, or cognitive state [41]. These results underscore the need for integrating rs-fMRI with more granular behavioral, psychological, and sensory phenotyping in future studies to better understand the clinical utility of functional biomarkers.

### Conclusion

The findings from our study underscore the significant potential of data-driven methodologies in unraveling the complex neural underpinnings of osteoarthritis (OA). Through advanced computational analyses, we identified pronounced atypical neural connections in key brain regions, including the cingulate gyrus, insula, and thalamus, which are deeply involved in pain and sensory processing in OA. Notably, we uncovered hidden connectivity patterns, such as the connections between the cingulate gyrus and regions like the temporal lobe, inferior parietal lobe, occipital lobe, and insula. Similarly, the thalamus exhibited concealed connections with the occipital lobe, insula, and basal ganglia. These previously unrecognized patterns reveal intricate relationships between regions that contribute to OA’s neurophysiological presentation.

The hub scores obtained in our analysis not only confirmed the central role of these regions within the atypical connectivity networks but also provided a quantitative framework to understand their relative importance in the disease process on an individual basis. This insight into hidden connectivity patterns reinforces the value of a data-driven approach in elucidating the neural architecture underlying OA. The success of this analytical approach in mapping and quantifying neural connectivity in OA suggests its broader applicability across a spectrum of neurological and systemic diseases. This method holds promise for uncovering concealed patterns and connections that are not readily apparent through traditional investigative techniques, serving as a powerful tool for diagnosis, treatment planning, and understanding diseases involving neural dysfunction. Furthermore, the scalability and adaptability of this data-driven approach enables its application in diverse clinical and research settings, potentially facilitating personalized medicine strategies and targeted therapies. By applying this methodology to other diseases, researchers and clinicians can develop more nuanced understandings of disease mechanisms, leading to more effective interventions tailored to the specific connectivity patterns of individual patients.

#### Limitations

A potential limitation of our study is the integration of longitudinal data from multiple clinical trials into a single cross-sectional framework. This approach may have introduced redundancy, as some OA patients underwent multiple assessments and could appear more than once in the dataset, potentially biasing the estimation of connectivity differences. Moreover, by pooling data from three distinct studies with different treatment arms, we may have inadvertently attenuated the detection of treatment-specific effects on brain connectivity. Future work should consider controlling for repeated measures or employing longitudinal modeling to better account for within-subject variability over time.

In addition, while our analysis included 56 OA patients and 20 healthy controls, each patient’s connectivity profile was compared individually against a normative distribution derived from the control group using permutation testing. This 1-vs-20 design supports individualized assessment and avoids traditional group-level imbalance issues. However, the relatively small control sample may limit the resolution of the null distributions and the generalizability of normative connectivity profiles. Future studies would benefit from expanding the healthy control dataset to improve both statistical robustness and representativeness.

Finally, our study did not collect real-time pain ratings during fMRI scanning, which limits our ability to fully dissociate connectivity patterns associated with chronic OA pathology from those reflecting momentary fluctuations in pain state. Incorporating concurrent pain assessments or longitudinal symptom tracking during scanning sessions would help clarify whether observed neural alterations reflect trait-like disease signatures or state-dependent changes in pain experience.

## Supplementary Files

This is a list of supplementary files associated with this preprint. Click to download.

- supplementaryInformation.docx

## Figures and Tables

**Table 1:** Demographics

	Osteoarthritis pain patients		Controls	
Gender	Male	Female	Male	Female
	26	30	10	10
Age (years)	57.6 ± 6.3	58.2 ± 7.6	57.8 ± 9.2	58.0 ± 3.1
VAS %	12.57 ± 25.53	22.12 ± 38.73*	NA	
WOMAC %	16.2 ± 28.8	21.1 ± 32.6	NA	

* *p*< 0.05, female group vs. male group (two-sample t-test).

## Data Availability

Availability of Data and MaterialsThe data used in this study are publicly available from the OpenNeuro repository under the accession number ds000208. The dataset can be accessed at: https://openneuro.org/datasets/ds000208
